# Gender Related Differences in the Clinical Presentation of Hypertrophic Cardiomyopathy—An Analysis from the SILICOFCM Database

**DOI:** 10.3390/medicina58020314

**Published:** 2022-02-18

**Authors:** Andrej Preveden, Miodrag Golubovic, Marija Bjelobrk, Tatjana Miljkovic, Aleksandra Ilic, Snezana Stojsic, Dragan Gajic, Mila Glavaski, Lars S. Maier, Nduka Okwose, Dejana Popovic, Fausto Barlocco, Arsen Ristic, Guy A. MacGowan, Iacopo Olivotto, Nenad Filipovic, Djordje G. Jakovljevic, Lazar Velicki

**Affiliations:** 1 Faculty of Medicine, University of Novi Sad, 21000 Novi Sad, Serbia; miodrag.golubovic@mf.uns.ac.rs (M.G.); marija.bjelobrk@mf.uns.ac.rs (M.B.); tajtana.miljkovic@mf.uns.ac.rs (T.M.); aleksandra.ilic@mf.uns.ac.rs (A.I.); milaglavaski85@yahoo.com (M.G.); lazar.velicki@mf.uns.ac.rs (L.V.); 2 Institute of Cardiovascular Diseases Vojvodina, 21204 Sremska Kamenica, Serbia; snezana.stojsic@ikvbv.ns.ac.rs (S.S.); gajic.dragan.91@gmail.com (D.G.); 3 Department of Internal Medicine II (Cardiology, Pneumology, and Intensive Care), University Medical Centre Regensburg, 93053 Regensburg, Germany; lars.maier@ukr.de; 4 Cardiovascular Research, Translational and Clinical Research and Biosciences Institute, Medicine, Newcastle University, Newcastle upon Tyne Hospitals NHS Foundation Trust, Newcastle upon Tyne NE2 4HH, UK; nduka.okwose@newcastle.ac.uk (N.O.); guy.macgowan@nhs.net (G.A.M.); djordje.jakovljevic@newcastle.ac.uk (D.G.J.); 5 Cardiovascular and Lifestyle Medicine Theme, Faculty Research Centre (CSELS), Institute for Health and Wellbeing, Faculty of Health Studies, Coventry University, University Hospital Coventry and Warwickshire, Coventry CV1 2TU, UK; 6 Clinical Center of Serbia, 11000 Belgrade, Serbia; dejana.popovic99@gmail.com (D.P.); arsen.ristic@gmail.com (A.R.); 7 Department of Experimental and Clinical Medicine, University of Florence, 50121 Florence, Italy; fausto.barlocco@unifi.it (F.B.); iacopo.olivotto@gmail.com (I.O.); 8 Bioengineering Research and Development Center, BioIRC, 34000 Kragujevac, Serbia; fica@kg.ac.rs; 9 Faculty of Engineering, University of Kragujevac, 34000 Kragujevac, Serbia

**Keywords:** hypertrophic cardiomyopathy, familial cardiomyopathy, left ventricular hypertrophy, hereditary cardiac disease, gender differences, systolic anterior motion

## Abstract

*Background and Objectives*: Hypertrophic cardiomyopathy (HCM) is the most common inherited cardiac disease that affects approximately 1 in 500 people. Due to an incomplete disease penetrance associated with numerous factors, HCM is not manifested in all carriers of genetic mutation. Although about two-thirds of patients are male, it seems that female gender is associated with more severe disease phenotype and worse prognosis. The objective of this study was to evaluate the gender related differences in HCM presentation. *Materials and Methods*: This study was conducted as a part of the international multidisciplinary SILICOFCM project. Clinical information, laboratory analyses, electrocardiography, echocardiography, and genetic testing data were collected for 362 HCM patients from four clinical centers (Florence, Newcastle, Novi Sad, and Regensburg). There were 33% female patients, and 67% male patients. *Results*: Female patients were older than males (64.5 vs. 53.5 years, *p* < 0.0005). The male predominance was present across all age groups until the age of 70, when gender distribution became comparable. Females had higher number of symptomatic individuals then males (69% vs. 52%, *p* = 0.003), most frequently complaining of dyspnea (50% vs. 30%), followed by chest pain (30% vs. 17%), fatigue (26% vs. 13%), palpitations (22% vs. 13%), and syncope (13% vs. 8%). The most common rhythm disorder was atrial fibrillation which was present in a similar number of females and males (19% vs. 13%, *p* = 0.218). Levels of N-terminal pro-brain natriuretic peptide were comparable between the genders (571 vs. 794 ng/L, *p* = 0.244). Echocardiography showed similar thickness of interventricular septum (18 vs. 16 mm, *p* = 0.121) and posterolateral wall (13 vs. 12 mm, *p* = 0.656), however, females had a lower number of systolic anterior motion (8% vs. 16%, *p* = 0.020) and other mitral valve abnormalities. *Conclusions*: Female patients are underrepresented but seem to have a more pronounced clinical presentation of HCM. Therefore, establishing gender specific diagnostic criteria for HCM should be considered.

## 1. Introduction

Hypertrophic cardiomyopathy (HCM) represents the most common monogenic inherited disease of the cardiovascular system that can be found in around 0.2% of the general population [1,2]. However, the clinical diagnosis is established in only one in every six patients with HCM, meaning that the majority of HCM patients are left undiagnosed during their lifetime and go under the radar of appropriate medical management [3].

The clinical diagnosis of HCM is based on left ventricular (LV) hypertrophy in the absence of cavity dilatation, which cannot be attributed to another cardiac, systemic, metabolic, or syndromic disease [2,4,5,6]. The course of HCM is very diverse, ranging from a completely asymptomatic, benign condition with a normal life expectancy to an advanced disease characterized by chest pain, dyspnea, heart failure, atrial fibrillation, malignant arrhythmia, syncope, or even sudden cardiac death [2,7]. Advanced stages of non-obstructive HCM can be and are mostly associated with increased myocardial fibrosis, microvascular ischemia, and abnormal cardiac function [8]. Novel findings suggest that HCM is associated with an increased oxidative stress and that markers of oxidative stress could even be used to identify patients with HCM [9].

HCM is caused by mutations in sarcomeric protein genes, which can involve the thick or thin filament or the Z-disc. The 2 most common mutations involve the thick filament—myosin-binding protein C (MYBPC3) and β-myosin heavy chain (MYH7), which are found in 75% of all HCM patients with identified mutations [10,11]. All mutations are transmitted in an autosomal dominant trait, meaning that HCM patients are generally heterozygotes, while each of their offspring have 50% chance of inheriting the genetic mutation. However, due to an incomplete disease penetrance associated with numerous factors, HCM is not manifested in all carriers of genetic mutation [12,13].

Among multiple factors involved, male sex stands out as one of the paramount determinants of HCM penetrance. The majority of HCM cohorts have heterogeneous gender distribution, with two thirds of patients being male [14,15,16,17]. In fact, male gender is associated with three times higher risk for developing HCM in mutation carriers [13]. On the other hand, despite the lower prevalence, female patients seem to have a more severe clinical presentation of the disease and a higher mortality rate [18,19].

Given the obvious differences in the characteristics of clinical expression of HCM between genders, the goal of this multicenter study was to investigate and further determine the influence of sex on clinical presentation and structural differences in the heart itself in HCM population.

## 2. Materials and Methods

As a part of the international multidisciplinary SILICOFCM project (silicofcm.eu) developing a computational platform for in silico clinical trials of familial cardiomyopathies, the present study evaluated the gender-related differences in patients with HCM.

The study protocol was approved by the UK National Health Service Health Research Authority North East-Tyne & Wear South Research Ethics Committee with the reference number 18/NE/0318 on 6 February 2019 and was adopted by the Institutional Review Board of each participating center. The study was conducted within the principles of Good Clinical Practice and following the Declaration of Helsinki.

### 2.1. Study Design

The study population consisted of 362 patients with HCM who underwent clinical evaluation at 1 of the 4 participating institutions: Newcastle University Medical School and Newcastle upon Tyne Hospitals NHS Foundation Trust (Newcastle, The United Kingdom), University Medical Centre Regensburg (Regensburg, Germany), Institute of Cardiovascular Diseases Vojvodina (Sremska Kamenica, Serbia), University of Belgrade Clinical Centre (Belgrade, Serbia), and Careggi University Hospital Florence (Florence, Italy). The collected data included demographic information, clinical characteristics, blood test results, 12-lead electrocardiography (ECG), echocardiography, and genetic testing results.

The diagnosis of HCM was defined according to the European Society of Cardiology guidelines i.e., maximal LV wall thickness of ≥15 mm (or ≥13 mm in individuals with positive family history of HCM), in the absence of any other cardiac or systemic disease that would be capable of producing LV hypertrophy, such as heart valve diseases or arterial hypertension [6]. Patients with significant atherosclerotic coronary artery disease (>50% stenosis in a major artery), prior cardiac surgery (including septal myectomy) and alcohol septal ablation, major LV outflow obstruction with pressure gradient > 50 mmHg, and chronic renal failure (creatinine clearance < 30 mL/min) were excluded from the study.

### 2.2. Echocardiography

To be included in the study, patients were required to have undergone transthoracic echocardiography examination. Images were acquired from standard parasternal and apical views, with simultaneous ECG monitoring. All of the parameters were calculated for the body surface area (BSA). LV wall thickness and chamber dimensions were measured in the parasternal long-axis view [20,21]. LV systolic and diastolic volumes and ejection fraction were calculated from Simpson’s modified biplane method from the apical 4-chamber and 2-chamber views [21]. Diastolic function was evaluated in the apical 4-chamber view [22]. Transmitral inflow was recorded using pulsed-wave Doppler at the tips of mitral valve leaflets. The peak velocity of the early diastolic filling (E) was measured. Early (e’s and e’l) and late (a’s and a’l) velocities of septal end lateral mitral annulus were measured using TDI and then their average ratio e’/a’ av was calculated. Numerous studies have shown that the E/e’, a volume-independent parameter, represents the most accurate index of the LV filling pressure [23]. This was calculated as the average ratio between E/e’s and E/e’l as E/e’ av.

### 2.3. Genetic Testing

Peripheral blood samples were acquired by phlebotomy. DNA was isolated using the QIAamp^®^ DNA Blood BioRobot MDx Kit (QIAGEN GmbH, Hilden, Germany). Genetic testing was performed using PCR technique/DNA sequencing. Blood samples were analyzed for the presence of mutations in the 8 most common causal genes for HCM encoding sarcomeric proteins. They represent the basis of the commonly available genetic panels for HCM. These are protein-coding genes responsible for encoding myosin-binding protein C (*MYBPC3*), thick-filament proteins (β-myosin heavy chain (*MYH7*) and the regulatory and essential light chains (*MYL2* and *MYL3*)), and thin-filament proteins (troponin T type 2 (*TNNT2*), troponin I type 3 (*TNNI3*), α-tropomyosin (*TPM1*), and α-actin (*ACTC*)).

### 2.4. Statistical Analysis

Continuous variables are expressed as mean values ± standard deviation for normally distributed data or median with interquartile range (IQR) (25th to 75th percentile) for non-normally distributed data, whereas categorical variables are presented as absolute numbers and percentages. The Kolmogorov-Smirnov test was used for the determination of quantitative data distribution. Mean values of continuous variables were compared using the independent samples *t*-test or Mann-Whitney U test, while categorical variables were compared using the chi-square test. Statistical significance for all of the tests was set at the *p* value of <0.05. All of the analyses were performed in SPSS version 20.0 (IBM SPSS Statistics, New York, NY, USA).

## 3. Results

### 3.1. General Characteristics

**Figure 1:**
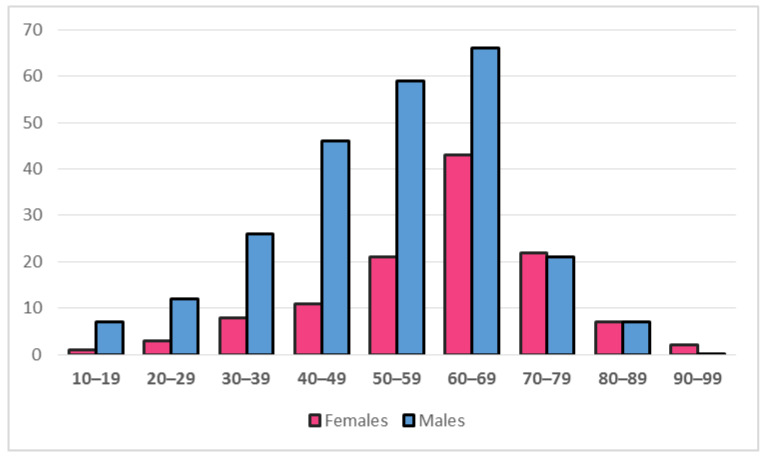
Age distribution of HCM patients according to gender. The difference in age distribution between genders is statistically significant (*p* = 0.002).

A total of 362 adult HCM patients from 4 clinical centers were included in the study. Gender distribution showed male predominance with 67.4%, compared to 32.6% females. Male to female ratio was 2:1. Female patients were significantly older than males (64.5 (IQR 54–70) vs. 53.5 (IQR 42–64) years, *p* < 0.0005). The male predominance was present across all age groups until the age of 70, when gender distribution became comparable (Figure 1). There was significant difference (*p* = 0.002) in the distribution of age groups between genders. In an isolated subpopulation of HCM patients above 50 years of age, the gender distribution was slightly more balanced, with 61.7% males and 38.3% females. The relative share of these patients in the overall population was different between genders: in male sex, individuals older than 50 years constituted 62.7% of the total group, while in female sex, they constituted 80.5% of the total group (Table 1). This difference in the distribution of HCM patients above 50 years of age within genders was statistically significant (*p* = 0.001), Body mass index was lower in females (25.9 (IQR 22.8–29.9) vs. 27.5 (IQR 24.5–30.0) kg/m^2^, *p* = 0.029), although both groups were slightly overweight.

### 3.2. Clinical Presentation

**Figure 2:**
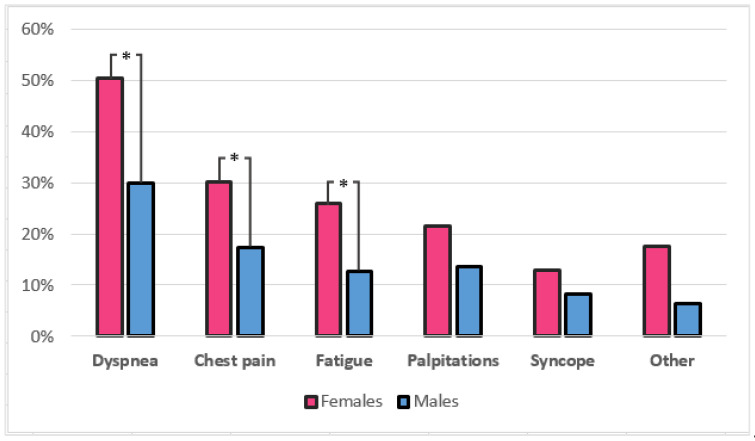
Prevalence of symptoms in HCM patients according to gender. * Differences are statistically significant (*p* < 0.05).

**Figure 3:**
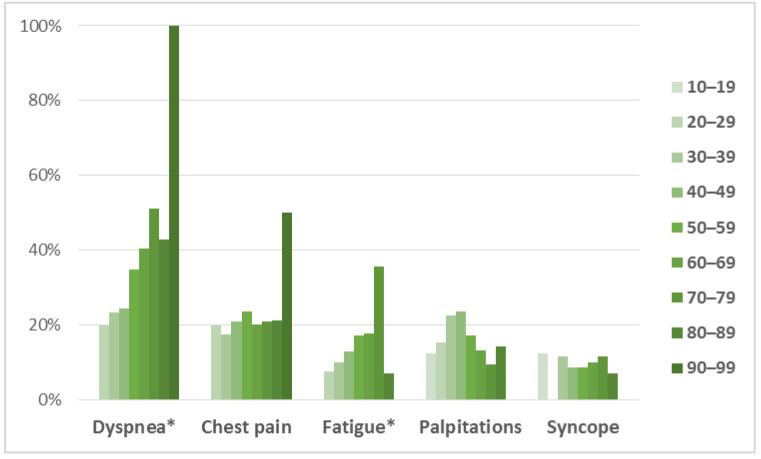
Prevalence of symptoms in HCM patients divided by age groups. * Difference in distribution is significant (*p* < 0.05).

Almost half of the male patients (48%) were asymptomatic, whereas only 1/3 of the female patients (31%) were free of symptoms (*p* = 0.003). In symptomatic patients, the most common complaint was dyspnea in both groups, followed by chest pain, fatigue, and palpitations, with least patients experiencing syncope (Table 1, Figure 2). All of the symptoms were more frequent in females, with significant difference observed for dyspnea (50.4% vs. 30.0%, *p* < 0.0005), chest pain (30.2% vs. 17.3%, *p* = 0.009), and fatigue (25.9% vs. 12.7%, *p* = 0.004). Prevalence of symptoms divided by age groups (Figure 3) showed significant difference in distribution for dyspnea (*p* = 0.007) and fatigue (*p* = 0.045), which displayed gradual increase in prevalence with age, while chest pain (*p* = 0.874), palpitations (0.666) and syncope (*p* = 0.960) were evenly distributed among age groups. The New York Heart Association (NYHA) functional class showed different distribution among genders (*p* = 0.007). Two-thirds (65.8%) of male patients belonged to NYHA class I who are free of heart failure related symptoms, compared to less than half (48.1%) of female patients. There were no patients with NYHA class IV.

The most common comorbidity was diabetes mellitus, which was similarly distributed in females and males (16.5% vs. 12.1%, *p* = 0.338). Thyroid disease was predominant in female patients (16.5% vs. 5.7%, *p* = 0.002), while all the other comorbidities had homogenous distribution among genders. Family history for HCM was positive in similar number of female (46.4%) and male (42.2%) patients. In contrast, females had higher number of sudden cardiac death reported in their family history compared to males (13.5% vs. 5.7%, *p* = 0.011). Heart rate, systolic and diastolic blood pressure were all within normal values and did not differ among groups.

### 3.3. Blood Laboratory Analyses

Electrolyte levels, markers of kidney and liver function were within normal range in all patients. When comparing groups, female patients had significantly lower levels of potassium, blood urea nitrogen and albumin. The values of N-terminal pro-brain natriuretic peptide (NT-proBNP) were elevated for both females (571 (IQR 173–1507) ng/L) and males (794 (IQR 372–1857) ng/L), however, no significant difference was observed between the groups.

### 3.4. Electrocardiography

Analysis of the 12-lead electrocardiography revealed that over three-quarters of patients in both groups had sinus rhythm (Table 2). The most common rhythm disorder was atrial fibrillation which was present in similar number of female and male patients (19.1% vs. 13.4%, *p* = 0.218). Importantly, females experienced higher number of paroxysmal supraventricular tachycardia (PSVT) than males (5.7% vs. 0.5%, *p* = 0.007). Sokolow-Lyon index, an ECG criterion for LV hypertrophy, showed normal values of <35 mm in both groups. Negative T waves were more frequent in female patients comparing to males (52.5% vs. 38.1%, *p* = 0.017). Left bundle branch block (LBBB) and right bundle branch block (RBBB) were equally distributed among genders.

### 3.5. Echocardiography

Although female patients demonstrated slightly higher LV wall thickness than males at both interventricular septum (18 (IQR 15–20) vs. 17 (IQR 15–21) mm, *p* = 0.121) and posterolateral wall (13 (IQR 11–14) vs. 12 (IQR 10–15) mm, *p* = 0.656), the difference was not statistically significant. Left atrial (LA) diameter and volume were smaller in females than males, as were left ventricular end-diastolic and end-systolic diameters (Table 3).

Systolic function of both ventricles was preserved in all patients. Left ventricular ejection fraction (LVEF) as a measure of LV systolic function was comparable in females and males (55 (IQR 55–66) vs. 60 (IQR 55–64) %, *p* = 0.672), and tricuspid annular plain systolic excursion (TAPSE) as a measure of right ventricle (RV) systolic function was also similar (22 (IQR 20–24) vs. 23 (IQR 20–26) mm, *p* = 0.436). Wall motion abnormalities were irregularly distributed among genders: males expressed higher rate of hypokinesia and akinesia, while females had more dyskinesia. Over half of the HCM patients had diastolic dysfunction, however, it was notably less frequent in females than males (40.7% vs. 61.5%, *p* < 0.0005).

Left ventricle outflow tract (LVOT) gradient was 11 mmHg higher in female patients (*p* < 0.0005), however, the number of individuals with LVOT gradient above 30 mmHg was comparable between females and males (11.0% vs. 6.1%, *p* = 0.104). Systolic anterior motion was more frequently registered in males (16.4%) compared to females (8.5%, *p* = 0.020), as were all other abnormalities of the mitral apparatus as well (Table 3). Surprisingly, myocardial fibrosis was registered only in male patients (5.3%).

### 3.6. Genetic Testing

**Figure 4:**
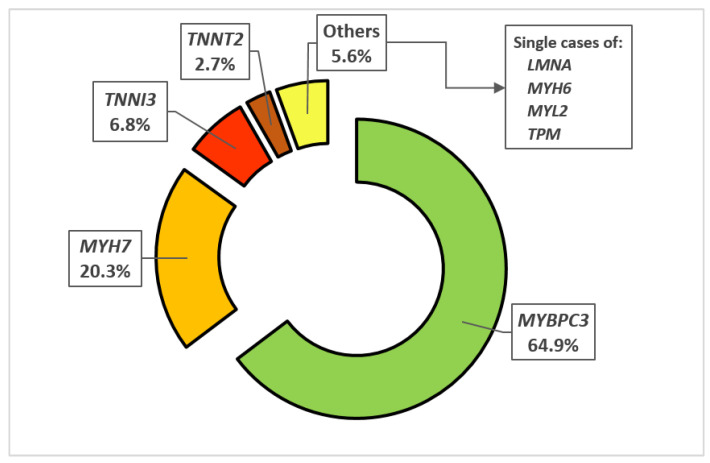
Frequency of specific gene mutations in HCM patients. Abbreviations: *MYBPC3*—myosin-binding protein C, *MYH6*—α-myosin heavy chain, *MYH7*—β-myosin heavy chain, *MYL2*—myosin light chain 2, *TNNI3*—troponin I type 3, *TNNT2*—troponin T type 2, *TPM*—α-tropomyosin.

Data on genetic testing was available for 148 (40.9%) patients. Genetic diagnosis of HCM (at least 1 mutation in the tested genes encoding sarcomeric proteins found) was confirmed in 107 out of 148 patients (72.3%) in whom genetic testing was conducted, of which 77 (72.0%) in males and 30 (28.0%) in females. The two dominating mutations were *MYBPC3* and *MYH7* that were discovered in 65.7% and 20.5%, respectively, while other mutations were found sporadically. The frequency of all of the genetic mutations is displayed in Figure 4. The distribution of genetic mutations was heterogeneous between genders (*p* = 0.033).

There were 73 patients with the genetic variant-level information in this study. From those, 7 patients (9.6%) had double mutations: 5 patients with 2 mutations in *MYBPC3*, 1 patient with mutations in *MYBPC3* and *MYH7*, and 1 patient with mutations in *MYBPC3* and *TPM1*. None of the 73 patients exhibited more than 2 mutations.

A total of 47 genetic variants were identified (12 of them were novel variants) in 73 patients (52 males and 21 females). Specifically, 35 out of 47 variants were already annotated in the dbSNP database, and among these: 11 were annotated as pathogenic, 9 as pathogenic/likely pathogenic, 3 as likely pathogenic, 6 as variants of uncertain significance, while 6 have conflicting interpretations of pathogenicity (Supplementary Table S1). Some variants were present in a number of patients. Pathogenic, pathogenic/likely pathogenic, and likely pathogenic variations were at total present in 36 (69.2%) males and 13 (61.9%) females.

## 4. Discussion

This multicenter study with HCM patients from four clinical centers across Europe investigated the differences in the presentation of HCM according to gender. The major findings of this study indicate that females with HCM are older and experience more symptoms with higher NYHA functional class for heart failure. While morphological and functional parameters assessed by echocardiography did show many distinctions, these findings were not convincing of either gender being affected by a more severe disease phenotype.

Gender related differences were more closely investigated in HCM for the first time more than two decades ago by Dimitrow et al. The group published several papers exploring differences in various aspects of HCM on the basis of sex. They were the first to perceive that female patients with HCM are significantly older than males, and also have delayed onset of symptoms [24]. Regardless of the somewhat worse clinical presentation, they showed that LV wall thickness does not differ between the genders [25], which is similar to our results, although other echocardiography parameters, such as LA and LV volumes and diameters were not analyzed. This was upgraded in the later studies [26,27] when the authors evaluated LV cavity sizes and showed that females with HCM have smaller LV cavity sizes, which is consistent with our results. The authors concluded that the smaller LV cavity, together with higher myocardial contractility, is predisposed to higher LVOT gradients in female HCM patients.

Our study group was dominated by men; women accounted for only 1/3 of the patients. Such gender distribution is consistent with other literature reports where females constitute between 25–45% of patients with HCM [18,19,28,29,30,31,32,33]. Given that HCM is inherited with an autosomal dominant pattern, a more balanced gender distribution would be expected. However, this is not the case due to reduced disease penetrance in women, as well as slower progression of myocardial hypertrophy that could be related to protective role of female sex hormones [34,35]. This theory is supported by our results, which show male predominance across all younger age groups, while above the age of 70 the gender distribution becomes equal (Figure 1). HCM is generally a disease of a younger age, as it is commonly first diagnosed around the age of 40 [28,36]. Our cohort of patients had a median age of 57 years at the time of clinical investigation, yet the women were 11 years older than men. Such a significant difference is consistent with the findings of other studies where this gap is reported to range from 6 to 13 years [19,28,29,32,37]. The underlying mechanisms for this obvious age discrepancy between genders remains incompletely understood. Possible explanations lie in the aforementioned protective role of female hormones which delay the phenotypic expression of HCM, combined with inadequate clinical recognition due to reduced awareness of cardiovascular risk in women [38], less indications for medical screening programs and clinician bias [39]. The theory of the loss of protective role of female hormones in menopausal women on the development of HCM is supported by the more balanced gender distribution in the population of HCM patients above 50 years of age, which is considered as usual age of menopause in average women [40]. In our cohort, the male to female ratio changes from 2:1 to 3:2 above the age of 50. In the male group, 1/3 of patients are younger than 50, while in the female group only 1/5 of patients are under 50. This implies that the vast majority of female mutation carriers develop HCM after the menopause and the following hormonal changes.

Furthermore, there are currently no gender specific criteria for the diagnosis of HCM [6,41]. This means that women require relatively higher level of hypertrophy in order to reach the diagnostic threshold of wall thickness >15 mm because of generally smaller heart size [34]. This could provide an explanation for a more severe clinical presentation in regard to more pronounced subjective symptoms such as shortness of breath, chest pain and palpitations, as well as higher NYHA functional class associated with female sex in our study, and consistently throughout the literature [18,28,29,32].

Although our study results did not show difference in LV wall thickness between the genders assessed by echocardiography, determinants of LA and LV cavity sizes were notably lower in females, supporting the argument of smaller hearts and higher level of relative hypertrophy observed in female HCM patients that are probably responsible for the differences in the severity of clinical presentation and outcomes. Consistently with other studies [42], our results demonstrate preserved systolic function of both ventricles in all of the patients. This finding supports the current standpoint that HCM generally does not lead to systolic dysfunction, but rather the clinical course is dominantly determined by the combination of diastolic dysfunction, mitral apparatus abnormalities, and LVOT obstruction [43,44]. Indeed, our results show that LVOT gradient was 11 mmHg higher in female patients, which contributed to the severity of HCM presentation. On the other hand, systolic anterior motion, and other mitral valve disorders, as well as myocardial fibrosis, were more frequently observed in males, which is in contrast with the findings from other studies [37,45].

A rising interest in the prognosis and survival of HCM patients has lately been observed. Several studies from various research groups with mixed population from across the globe tried to determine whether gender has an influence on the outcome of HCM. Olivotto et al. [28], in their study from 2005, examined gender-related differences in a population of 969 patients with HCM. Similarly to our results, male patients had a 59% predominance. They also pointed out that women with HCM were under-represented, older, and more symptomatic than men, and also showed a higher risk of progression to advanced heart failure or death, often associated with LVOT obstruction. However, their main results, similar to the results of Rowin et al. [37] who included a population of 2123 HCM patients, did not show any differences in survival rates, HCM-related mortality and risk of sudden death between the sexes. Moreover, 2 of the latest studies by Kim et al. [33] and Lakdawala et al. [32] are the largest studies with HCM patients so far, which included 9524 and 5873 patients, respectively. Both studies found that women with HCM had poorer prognosis than men, which is mainly attributed to the higher rate of heart failure-related hospitalizations. Despite the obvious higher burden that females with HCM carry for worsening symptoms requiring medical attention and healthcare visits, difference in mortality rates between the sexes in HCM has not yet been conclusively determined, ranging from completely balanced [33,37] to significantly higher in females [19,32,46] in various studies.

In our previous study from the SILICOFCM database [16], we investigated the genetic determinacy of various clinical phenotype parameters among patients with HCM. Only the carriers of a single gene mutation, in either *MYBPC3* or *MYH7* were included. This study found that *MYH7* gene mutation causes a slightly more severe clinical phenotype of HCM with higher LV filling pressures and higher rate of mitral valve abnormalities including systolic anterior motion. Interestingly, gender distribution was not homogenous between the groups: patients with *MYH7* gene mutation were 33.3% female, and patients with *MYBPC3* gene mutation were only 20.8% female. This uneven distribution of the gene mutations across male and female patients with HCM could provide an explanation of the genetic basis for the different presentation and disease severity between the genders. Interestingly, although men are consistently younger than women in joined HCM cohorts, *MYH7* gene mutations represents an exception where both genders are of similar age [32]. This fact suggests that gender does not modify the penetrance of HCM for *MYH7* gene mutation in contrast to other sarcomeric gene mutations, primarily the most common *MYBPC3* gene mutation.

In perspective, we hope that our results will contribute to the overall understanding of the underlying mechanisms in the development of HCM and strengthen the basis for establishing a more individually oriented HCM management. The results of this study contributed to the creation of the concept and design of the multicenter SILICOFCM trial [47,48], which seeks to provide novel data on whether the complementary addition of either sacubitril/valsartan or lifestyle intervention to the optimal standard therapy improves cardiovascular performance in patients with non-obstructive HCM as well as their clinical phenotypic characteristics, injury and stretch activation markers, habitual physical activity, and quality of life.

## 5. Study Limitations

Although our study showed that females with HCM were significantly older than males, we based this finding only on the information about the patients’ age at the moment of clinical evaluation. The age at initial HCM diagnosis could have contributed to more detailed analysis regarding differences in disease onset and duration and provide insight into the details about the delay in clinical recognition of HCM in female patients.

The large number of operators involved in echocardiographic measurements in this multicenter study represents an unavoidable limitation. This fact could provide an explanation for the somewhat contrasting findings of disease severity parameters such as diastolic dysfunction, LVOT obstruction, mitral valve abnormalities and myocardial fibrosis. However, care was taken to standardize measurements of cardiac dimension and function by prospectively providing detailed technical instructions to all of the participating centers.

Unfortunately, genetic testing results were available for less than half of our patients, so the genetic basis of the gender related differences could not be determined with certainty. A more comprehensive genetic analysis is required to find a link between specific gene mutations and severity of phenotypic expression among genders. In this study, the presence of mutations in the 8 most common causal genes for HCM encoding sarcomeric proteins was determined. However, other genes, not investigated, may be responsible for the more pronounced clinical presentation of HCM in female subjects. Moreover, there was 9.6% of patients in this study who exhibited 2 HCM causing mutations. Since patients who exhibit two causal mutations in same or different genes present more severe phenotype [49,50], this might have affected the presented results as well.

## 6. Conclusions

Female patients are underrepresented but seem to have a more pronounced clinical presentation of HCM. Therefore, establishing gender specific diagnostic criteria for HCM should be considered.

## Figures and Tables

**Table 1:** Clinical characteristics of HCM patients according to gender.

	Overall	Females	Males	*p* Value
Number of patients	362 (100.0%)	118 (32.6%)	244 (67.4%)	
Age (years)	57 (46–67)	64.5 (54–70)	53.5 (42–64)	<0.0005 *
Age ≥ 50 years	248 (68.5%)	95 (80.5%)	153 (62.7%)	0.001 *
Body mass index (kg/m^2^)	27.2 (23.9–30.0)	25.9 (22.8–29.9)	27.5 (24.5–30.0)	0.029 *
Symptoms				
Fatigue	59 (17.2%)	30 (25.9%)	29 (12.7%)	0.004 *
Dyspnea	127 (36.8%)	58 (50.4%)	69 (30.0%)	<0.0005 *
Chest pain	75 (21.6%)	35 (30.2%)	40 (17.3%)	0.009 *
Palpitations	56 (16.2%)	25 (21.6%)	31 (13.5%)	0.077
Syncope	34 (9.8%)	15 (12.9%)	19 (8.2%)	0.230
Other	15 (10.4%)	9 (17.6%)	6 (6.5%)	0.047 *
NYHA classification				
I	183 (59.8%)	50 (48.1%)	133 (65.8%)	0.007 *
II	96 (31.4%)	40 (38.5%)	56 (27.7%)	
III	27 (8.8%)	14 (13.5%)	13 (6.4%)	
Comorbidities				
Diabetes mellitus	47 (13.6%)	19 (16.5%)	28 (12.1%)	0.338
Thyroid disease	32 (9.3%)	19 (16.5%)	13 (5.7%)	0.002 *
Renal dysfunction	16 (4.7%)	8 (7.0%)	8 (3.5%)	0.177
Hepatic dysfunction	3 (0.9%)	1 (0.9%)	2 (0.9%)	1.000
COPD	16 (4.6%)	5 (4.3%)	11 (4.8%)	1.000
Anemia	7 (1.9%)	4 (3.4%)	3 (1.2%)	0.162
Neuromuscular disease	3 (0.9%)	2 (1.8%)	1 (0.4%)	0.253
Positive family history				
HCM	72 (43.6%)	26 (46.4%)	46 (42.2%)	0.724
Sudden cardiac death	30 (8.3%)	16 (13.5%)	14 (5.7%)	0.011 *
Vital signs				
Systolic blood pressure (mmHg)	120 (110–140)	120 (110–140)	123 (115–140)	0.169
Diastolic blood pressure (mmHg)	78 (70–80)	70 (67–82.5)	80 (70–80)	0.052
Heart rate (beats per minute)	64 (57–73)	64 (59–75)	63 (57–70)	0.203
Laboratory results				
Sodium (mmol/L)	140 (193–142)	140 (193–142)	140 (193–142)	0.920
Potassium (mmol/L)	4.2 (4.0–4.5)	4.1 (3.9–4.3)	4.3 (4.0–4.6)	0.001 *
Blood urea nitrogen (mmol/L)	6.4 (4.9–9.6)	5.7 (4.2–6.8)	7.2 (5.3–10.7)	<0.0005 *
Creatinine (µmol/L)	82 (70–98)	80 (66–94)	82 (71–102)	0.176
Uric acid (µmol/L)	352 (287–417)	355 (277–415)	351 (294–416)	0.980
ALT (U/L)	27 (19–39)	30 (21–39)	26 (19–37)	0.151
AST (U/L)	23 (18–33)	25 (17–39)	23 (18–31)	0.137
LDH (U/L)	216 (176–275)	222 (196–283)	209 (169–267)	0.092
Total protein (g/L)	72 (67–76)	75 (69–78)	71 (67–75)	0.064
Albumin (g/L)	42 (36–45)	40 (36–43)	46 (44–49)	<0.0005 *
Creatine kinase (U/L)	105 (67–145)	111 (71–164)	104 (66–134)	0.479
LDL cholesterol (mmol/L)	3.1 (±1.0)	3.0 (±1.0)	3.3 (±1.0)	0.129
HDL cholesterol (mmol/L)	1.2 (1.0–1.5)	1.3 (1.1–1.5)	1.2 (1.0–1.5)	0.258
NT-proBNP (ng/L)	728 (291–1789)	571 (173–1507)	794 (372–1857)	0.244

Categorical variables are shown as n (%); continuous variables are shown as median (interquartile range) or mean (± standard deviation) depending on data distribution normality. * Differences are statistically significant (*p* < 0.05). Abbreviations: ALT—alanine transaminase, AST—aspartate transaminase, COPD—chronic obstructive pulmonary disease, HCM—hypertrophic cardiomyopathy, HDL—high-density lipoprotein, LDH—lactate dehydrogenase, LDL—low-density lipoprotein, NYHA—New York Heart Association, NT-proBNP—N-terminal pro-brain natriuretic peptide.

**Table 2:** ECG findings in HCM patients according to gender.

KERRYPNX	Overall	Females	Males	*p* Value
Sinus rhythm	277 (78.5%)	87 (75.7%)	190 (79.8%)	0.449
Atrial flutter	11 (3.1%)	4 (3.5%)	7 (2.9%)	0.753
Atrial fibrillation	54 (15.3%)	22 (19.1%)	32 (13.4%)	0.218
PSVT	7 (2.3%)	6 (5.7%)	1 (0.5%)	0.007 *
Ventricular tachycardia	25 (8.1%)	7 (6.7%)	18 (8.9%)	0.661
PR interval (ms)	182 (160–206)	177 (160–200)	184 (161–207)	0.206
QRS duration (ms)	108 (94–127)	106 (90–132)	108 (96–124)	0.448
Sokolow-Lyon index (mm)	30 (22–37)	26 (21–35)	32 (25–38)	0.166
Significant Q waves	47 (13.0%)	13 (11.0%)	34 (13.4%)	0.342
ST segment abnormalities	85 (23.5%)	34 (28.8%)	51 (20.9%)	0.096
Negative T waves	155 (42.8%)	62 (52.5%)	93 (38.1%)	0.017 *
LBBB	31 (8.6%)	12 (10.2%)	19 (7.8%)	0.448
RBBB	32 (8.8%)	12 (10.2%)	20 (8.2%)	0.535

Categorical variables are shown as n (%); continuous variables shown as median (interquartile range). * Differences are statistically significant (*p* < 0.05). Abbreviations: LBBB—left bundle branch block, PSVT—paroxysmal supraventricular tachycardia, RBBB—right bundle branch block.

**Table 3:** Echocardiography parameters in HCM patients according to gender.

	Overall	Females	Males	*p* Value
Interventicular septum thickness (mm)	17 (15–21)	18 (15–20)	17 (15–21)	0.121
Posterolateral wall thickness (mm)	13 (10–15)	13 (11–14)	12 (10–15)	0.656
LA diameter (mm)	43 (39–49)	34 (32–37)	44 (39–49)	<0.0005 *
LA volume (ml)	82 (59–107)	54 (35–67)	85 (64–110)	<0.0005 *
LA volume/BSA (ml/m^2^)	39 (27–52)	28 (20–36)	40 (28–53)	0.003 *
LVEDD (mm)	47 (42–51)	45 (42–50)	48 (44–53)	0.009 *
LVESD (mm)	28.6 (±7.4)	26.5 (±6.1)	29.2 (±7.7)	0.036 *
LVEF (%)	60 (55–65)	55 (55–66)	60 (55–64)	0.672
E/E’ ratio	10 (8–14)	11 (8–14)	10 (8–14)	0.900
Diastolic dysfunction	198 (54.7%)	48 (40.7%)	150 (61.5%)	<0.0005 *
LVOT gradient (mmHg)	9 (5–15)	18 (10–36)	7 (5–13)	<0.0005 *
LVOT gradient >30 mmHg	28 (7.7%)	13 (11.0%)	15 (6.1%)	0.104
Mitral valve abnormalities				0.020 *
Systolic anterior motion	50 (13.8%)	10 (8.5 %)	40 (16.4%)	
Papillary muscle abnormalities	13 (3.6%)	0 (0.0%)	13 (5.3%)	0.005 *
Mitral leaflet abnormalities	80 (22.1%)	4 (3.4 %)	76 (31.1%)	<0.0005 *
Calcification of mitral annulus	28 (7.7%)	1 (0.8%)	27 (11.1%)	<0.0005 *
Myocardial fibrosis	13 (3.6%)	0 (0.0%)	13 (5.3%)	0.006 *
Wall motion abnormalities				
Hypokinesia	50 (13.8%)	1 (0.8%)	49 (20.1%)	<0.0005 *
Akinesia	14 (3.9%)	1 (0.8%)	13 (5.3%)	0.014 *
Dyskinesia	60 (16.6%)	47 (39.8%)	13 (5.3%)	<0.0005 *
TAPSE (mm)	22 (20–26)	22 (20–24)	23 (20–26)	0.436

Categorical variables are shown as n (%); continuous variables shown as median (interquartile range) or mean (± standard deviation) depending on data distribution normality. * Differences are statistically significant (*p* < 0.05). Abbreviations: BSA—body surface area, LA—left atrium, LVEF—left ventricle ejection fraction, LVEDD left ventricle end-diastolic dimension, LVESD—left ventricle end-systolic dimension, LVOT—left ventricle outflow tract, TAPSE—tricuspid annular plain systolic excursion.

## Data Availability

The data presented in this study are available on reasonable request from the corresponding author.

## Supplementary Materials

The following supporting information can be downloaded at: https://www.mdpi.com/article/10.3390/medicina58020314/s1, Table S1: List of detected variants in HCM patients.
