# Supplementary material for: Gender Related Differences in the Clinical Presentation of Hypertrophic Cardiomyopathy—An Analysis from the SILICOFCM Database
Source: Medicina (Kaunas). 2022 Feb 18;58(2):314. doi: 10.3390/medicina58020314 (PMC8879033; doi:10.3390/medicina58020314)
Supplement: Supplementary file 1 [file medicina-58-00314-s001.zip › medicina-1570138-supplementary.pdf]

Supplementary Table S1. List of detected variants in HCM patients

Mutated gene	Genetic variant<br><br>Coding DNA reference sequence	Genetic variant<br><br>Protein reference sequence	Reference SNP ID	Consequence	Clinical significance by ClinVar database	Sex of the patient
MYBPC3	c.3192dup	p.Lys1065Glnfs*12	rs397516007	Frameshift Variant	Pathogenic	Male
MYBPC3	c.1624G>C	p.Glu542Gln	rs121909374	Stop Gained	Pathogenic	Male
MYBPC3	c.1615A>G	p.Ile539Val	-	-	-	Male
MYBPC3	c.772C>A	p.Glu258Lys	-	-	-	Male
MYBPC3	c.772G>A	p.Glu258Lys	rs397516074	Missense Variant	Pathogenic/Likely pathogenic	Male
MYBPC3	c.2398G>A	p.Gly800Arg	rs727504574	Missense Variant	Uncertain significance	Male
MYBPC3	c.2846dup	p.Met949Ilefs*102	-	-	-	Male
MYBPC3	c.1624G>C	p.Glu542Gln	rs121909374	Stop Gained	Pathogenic	Male
MYBPC3	c.1458-1G>A	p.?	rs397515903	Splice Acceptor Variant	Pathogenic	Male
MYBPC3	c.772G>A	p.Glu258Lys	rs397516074	Missense Variant	Pathogenic/Likely pathogenic	Male
MYBPC3	c.2689_2698del	p.Gly897Alafs*24	-	-	-	Male
MYBPC3	c.407-1G>A	p.?	-	-	-	Male
MYBPC3	c.1591G>C	p.Gly531Arg	rs397515912	Missense Variant	Likely pathogenic	Male
MYBPC3	c.1591G>C	p.Gly531Arg	rs397515912	Missense Variant	Likely pathogenic	Male
MYBPC3	c.913_914del	p.Phe305Profs*27	rs397516080	Frameshift Variant	Pathogenic/Likely pathogenic	Male
MYBPC3	c.1020C>G	p.Tyr340*	-	-	-	Male
MYBPC3	c.1505G>A	p.Arg502Gln	rs397515907	Missense Variant	Pathogenic/Likely pathogenic	Male
MYBPC3	c.772G>A	p.Glu258Lys	rs397516074	Missense Variant	Pathogenic/Likely pathogenic	Male
MYBPC3	c.2728C>A	p.Pro910Thr	rs397515985	Missense Variant	Conflicting interpretations of pathogenicity<br>Likely benign(1);Uncertain significance(9)	Male
MYBPC3	c.772G>A	p. Glu258Lys	rs397516074	Missense Variant	Pathogenic/Likely pathogenic	Male
MYBPC3	c.772G>A	p. Glu258Lys	rs397516074	Missense Variant	Pathogenic/Likely pathogenic	Male
MYBPC3	c.913_914del	p.Phe305Profs*27	rs397516080	Frameshift Variant	Pathogenic/Likely pathogenic	Male
MYBPC3	c.3192dup	p.Lys1065Glnfs*12	rs397516007	Frameshift Variant	Pathogenic	Male

MYBPC3	c.1112C>G	p.Pro371Arg	rs397515887	Missense Variant	Uncertain significance	Male
MYBPC3	c.3192dup	p.Lys1065Glnfs*12	rs397516007	Frameshift Variant	Pathogenic	Male
MYBPC3	c.1504C>T	p.Arg502Trp	rs375882485	Missense Variant	Conflicting interpretations of pathogenicity Likely pathogenic(1);Pathogenic(14);Uncertain significance(2)	Male
MYBPC3	c.1504C>T	p.Arg502Trp	rs375882485	Missense Variant	Conflicting interpretations of pathogenicity Likely pathogenic(1);Pathogenic(14);Uncertain significance(2)	Male
MYBPC3	c.1504C>T	p.Arg502Trp	rs375882485	Missense Variant	Conflicting interpretations of pathogenicity Likely pathogenic(1);Pathogenic(14);Uncertain significance(2)	Male
MYBPC3	c.1504C>T	p.Arg502Trp	rs375882485	Missense Variant	Conflicting interpretations of pathogenicity Likely pathogenic(1);Pathogenic(14);Uncertain significance(2)	Male
MYBPC3	c.1504C>T	p.Arg502Trp	rs375882485	Missense Variant	Conflicting interpretations of pathogenicity Likely pathogenic(1);Pathogenic(14);Uncertain significance(2)	Male
MYBPC3	c.1928-2A>G	p.?	rs397515937	Splice Acceptor Variant	Pathogenic	Male
MYBPC3	c.2441_2443del	p.Lys814del	rs727504288	Inframe Deletion	Conflicting interpretations of pathogenicity Likely pathogenic(2);Uncertain significance(10)	Male
MYBPC3	c.3226_3227insT	p.Asp1076fs	rs397516008	Frameshift Variant	Pathogenic	Male

MYBPC3	c.3226_3227insT	p.Asp1076fs	rs397516008	Frameshift Variant	Pathogenic	Male
MYBPC3	c.517_519del	p.?	-	-	-	Male
MYBPC3	c.655G>C	p.Val219Leu	rs397516068	Missense Variant	Pathogenic/Likely pathogenic	Male
MYBPC3	c.655G>C	p.Val219Leu	rs397516068	Missense Variant	Pathogenic/Likely pathogenic	Male
MYBPC3	c.772G>A	p.Glu258Lys	rs397516074	Missense Variant	Pathogenic/Likely pathogenic	Male
MYBPC3	c.772G>A	p.Glu258Lys	rs397516074	Missense Variant	Pathogenic/Likely pathogenic	Male
MYBPC3	c.772G>A	p.Glu258Lys	rs397516074	Missense Variant	Pathogenic/Likely pathogenic	Male
MYBPC3	c.772G>A	p.Glu258Lys	rs397516074	Missense Variant	Pathogenic/Likely pathogenic	Male
MYH7	c.2346C>A	p.Ser782Arg	rs730880736	Missense Variant	Likely pathogenic	Male
MYH7	c.2302G>A	p.Gly768Arg	rs727503260	Missense Variant	Pathogenic/Likely pathogenic	Male
MYH7	c.2207T>C	p.Ile736Thr	rs727503261	Missense Variant	Pathogenic	Male
MYH7	c.2389G>A	p.Ala797Thr	rs3218716	Missense Variant	Pathogenic/Likely pathogenic	Male
MYH7	c.2722C>G	p.Leu908Val	rs121913631	Missense Variant	Pathogenic	Male
MYH7	c.2722C>G	p.Leu908Val	rs121913631	Missense Variant	Pathogenic	Male
MYH7	c.2722C>G	p.Leu908Val	rs121913631	Missense Variant	Pathogenic	Male
MYH7	c.715G>A	p.Asp239Asn	rs397516264	Missense Variant	Likely pathogenic	Male
MYH7	c.715G>A	p.Asp239Asn	rs397516264	Missense Variant	Likely pathogenic	Male
MYH7	c.715G>A	p.Asp239Asn	rs397516264	Missense Variant	Likely pathogenic	Male
TNNT2	c.517_519del	p.Glu173del	-	-	-	Male
TNNI3	c.557G>A	p.Arg186Gln	rs397516357	Missense Variant	Pathogenic/Likely pathogenic	Male
TNNI3	c.625G>A	p.Glu209Lys	rs727504268	Missense Variant	Uncertain significance	Male
TPM1	c.4871C>T	p.?	-	-	-	Male
MYL2	c.58A>C	p.Met20Leu	-	-	-	Male
MYBPC3	c.1174del	p.Ala392Leufs*14	rs1565628486	Frameshift Variant	Pathogenic	Female
MYBPC3	c.1789C>T	p.Arg597Trp	rs201596087	Missense Variant	Uncertain significance	Female
MYBPC3	c.1090G>A	p.Ala364Thr	rs794727046	Missense Variant	Pathogenic/Likely pathogenic	Female

TPM1	c.375-3C>T	p.?	rs202228866	Intron Variant	Conflicting interpretations of pathogenicity Benign(5);Likely benign(2);Uncertain significance(2)	Female
MYBPC3	c.1458-1G>A	p.?	rs397515903	Splice Acceptor Variant	Pathogenic	Female
MYBPC3	c.772G>A	p.Glu258Lys	rs397516074	Missense Variant	Pathogenic/Likely pathogenic	Female
MYBPC3	c.2429G>T	p.Arg810Leu	rs375675796	Missense Variant	Conflicting interpretations of pathogenicity Likely pathogenic(3);Uncertain significance(1)	Female
MYBPC3	c.1224-52G>A	p.?	rs786204336	Intron variant	Pathogenic/Likely pathogenic	Female
MYBPC3	c.1504C>T	p.Arg502Trp	rs375882485	Missense Variant	Conflicting interpretations of pathogenicity Likely pathogenic(1);Pathogenic(14);Uncertain significance(2)	Female
MYBPC3	c.1504C>T	p.Arg502Trp	rs375882485	Missense Variant	Conflicting interpretations of pathogenicity Likely pathogenic(1);Pathogenic(14);Uncertain significance(2)	Female
MYBPC3	c.2373dupG	p.Trp792Valfs	rs397515963	Frameshift Variant	Pathogenic	Female
MYBPC3	c.3065G>C	p.Arg1022Pro	rs397516000	Missense Variant	Conflicting interpretations of pathogenicity Likely pathogenic(3);Pathogenic(1);Uncertain significance(4)	Female
MYH7	c.4130C>T	p.Thr1377Met	rs397516201	Missense Variant	Pathogenic	Female
MYH7	c.2167C>T	p.Arg723Cys	rs121913630	Missense Variant	Pathogenic	Female
MYH7	c.2167C>T	p.Arg723Cys	rs121913630	Missense Variant	Pathogenic	Female
MYH7	c.715G>A	p.Asp239Asn	rs397516264	Missense Variant	Likely pathogenic	Female
MYBPC3	c*26+2T>C	p.?	-	-	-	Female

MYH7	c.715G>A	p.Asp239Asn	rs397516264	Missense Variant	Likely pathogenic	Female
TNNT2	c.274C>T	p.Arg92Trp	-	-	-	Female
TNNI3	c.557G>A	p.Arg186Gln	rs397516357	Missense Variant	Pathogenic/Likely pathogenic	Female
TNNI3	c.557G>A	p.Arg186Gln	rs397516357	Missense Variant	Pathogenic/Likely pathogenic	Female
TNNI3	c.511G>A	p.Ala171Thr	rs121917761	Missense Variant	Uncertain significance	Female
MYH6	c.5519A>G	p.Lys1840Arg	rs373629059	Missense Variant	Uncertain significance	Female

Dashed line indicates that two variants (separated by the dashed line) are found in the same patient.
